# Added value of brain perfusion ultrasound to prehospital stroke scales-a secondary analysis of the SONAS trial

**DOI:** 10.3389/fneur.2026.1924664

**Published:** 2026-10-23

**Authors:** Nicole Birner, Sibylle Wilfling, Mustafa Kilic, Christina Wendl, Amir M. Molaie, Ralf A. Linker, David S. Liebeskind, Felix Schlachetzki

**Affiliations:** 1 Department of Neurology, University of Regensburg, Medbo Bezirksklinikum, TEMPiS Telestroke Center, and University Clinic Regensburg, Regensburg, Germany; 2 Department of Anaesthesiology, St. Josef Hospital Regensburg, Regensburg, Germany; 3 Center for Human Genetics, Regensburg, Germany; 4 Center for Neuroradiology, University Clinic Regensburg, Regensburg, Germany; 5 Department of Neurology, University of California, Los Angeles Medical Center, Los Angeles, CA, United States

**Keywords:** brain perfusion ultrasound, large vessel occlusion, mechanical thrombectomy (MT), prehosptial stroke diagnostics, SONAS, stroke

## Abstract

**Introduction:**

Accurate prehospital identification of acute ischemic stroke due to large vessel occlusion (LVO) is critical to guide selection of an optimal target hospital for either intravenous thrombolysis (IVT) alone and/or mechanical thrombectomy (MT). The novel portable non-imaging brain perfusion ultrasound (BPU) device SONAS(TM) indicated similar sensitivity and specificity as prehospital stroke scales. In this study, we reanalyzed data from the original 20 patient cohort to evaluate whether combining BPU with prehospital stroke scales (ph-SS) can further improve LVO detection.

**Methods:**

Clinical data and NIHSS scores of all patients suffering from acute ischemic stroke with and without proven LVO in the SONAS trial were collected and applied to 16 different phSS. As an exploratory analysis we also applied a prehospital score for intracerebral hemorrhage (ph-ICH) to this cohort. The positive and negative predictive values as well as sensitivity and specificity were analyzed retrospectively for both methods.

**Results:**

The best performing phSS, CG-FAST, and RACE, showed comparable performances to BPU, but still misdiagnosed patients. However, in our specific cohort, the combination of both methods allowed for a reliable classification into three groups: (A) “highly probably LVO”, (B) “highly probable non-LVO” and (C) “not assessable” (including all patients misdiagnosed by one method alone). The addition of BPU resulted in shifts towards higher sensitivity and NPV. When using the ph-ICH score three patients were classified as ICH (false positive), and all of them showed severe perfusion deficits in BPU indicating LVO.

**Conclusion:**

The combination of BPU with phSS may hold the potential to enhance prehospital triage of acute stroke patients selecting patients to (A) endovascular-capable centers, (B) the nearest stroke unit for, i.e., IVT or (C) either depending on other clinical factors and local circumstances. However, validation is critical in stroke-call patients in the prehospital setting including application by paramedics with/without emergency physicians or telemedical support, and compared to other prehospital point-of-care diagnostics both in respect to sensitivity/specificity, timeline and, ultimately, costs. In the future, the addition of serum biomarkers for ICH may open new opportunities for early ICH treatment or even enable prehospital IVT, and/or streamline direct to angio-suite approaches in confirmed LVO.

## Introduction

Stroke is the second leading cause of death worldwide (1, 2). Due to advances in cardiovascular medicine and in recanalization therapies, including intravenous thrombolysis (IVT) and especially mechanical thrombectomy (MT, synonymous: endovascular thrombectomy, EVT) for large vessel occlusion (LVO), the number of strokes and stroke survivors has increased over the past 2 ½ decades (3–5).

Despite the aforementioned advances, acute ischemic stroke therapy remains a time-critical disease, and comprehensive stroke centers (CSC) capable of EVT are not evenly distributed, particularly in rural areas (6, 7). While telestroke networks enable a framework for IVT, patients often need to be transferred for EVT, and in some instances, interventionalists are even transferred for MT by car, helicopter or even plane (8–10). Yet, secondary transport of patients for MT is time-consuming and can worsen the patient’s prognosis (11, 12). LVO detection in the prehospital setting and direct transfer to CSCs can lead to faster symptom-to-recanalization times. In the Maryland stroke study, preclinical triage strategies for LVO detection indeed led to faster transport to a CSC which translated to improved outcomes by avoiding delayed transport to a closer primary stroke center (PSC) (i.e., “drip and ship”), however, as only patients with LVO were analyzed and not the whole stroke population including IVT only a selection bias exists (13, 14). This was done without disproportionately delaying IVT, yet the effect of overtriage for the CSCs by false-positive LVO patients may vary regionally (4, 7, 8, 14, 15).

Although many clinical stroke scores have been published to identify LVO (16–24), very few of them have been validated “in the field” by paramedics, and even fewer amongst patients with acute ischemic stroke eligible for intravenous thrombolysis (IVT), hemorrhagic stroke, or stroke mimics (18, 25–27). The sensitivity of these scores is significantly inferior to the specificity, with acceptable to good results (28). No scale has both high sensitivity and specificity for determining large vessel occlusion, so that despite a negative test, a LVO may still be present with a probability of ≥10% (29). However, in the absence of mobile CT or other brain imaging tools, all guidelines worldwide recommend the use of stroke scales, though none endorse a specific scale as having clearly superior performance (30). When prehospital scores alone are used to diagnose LVO and decide on a target hospital, 50% of patients are inappropriately diverted to a CSC, causing potential harm by delaying IVT (13). Therefore, a method with higher sensitivity would be optimal as an addition to the established scales.

Several strategies are currently applied or in the research phase: mobile stroke units, stroke serum biomarkers, electroencephalography (EEG) and near infrared spectro-scopy, amongst others. Neurovascular ultrasound such as transcranial Doppler (TCD) or transcranial color-coded sonography (TCCS) is uniquely suited for the prehospital setting, offering a mobile, non-invasive, relatively inexpensive solution that can be guided by telemedicine and is aimed directly at diagnosing LVO. In the Regensburg stroke mobile project, stroke neurologists joined the emergency team at the patient’s site and applied TCCS in conjunction with echo-enhancing agents (EEA). The combination of TCCS with neurological stroke knowledge had a sensitivity of 90% and specificity of 98% for middle cerebral artery occlusion (MCAO) (31, 32). In contrast to CT, TCCS is pathophysiologically oriented; for example, in a case of left sided hemiparesis, the investigation starts at the contralateral skull for right MCAO detection.

A novel mobile sonography device was recently developed, specifically designed to investigate hemispheric bilateral brain perfusion (SONAS™) as a point-of-care (POC) diagnostic. A phase II-III study on brain perfusion ultrasound (BPU) in pre-selected acute ischemic stroke patients who underwent CT-perfusion indicated a sensitivity of 93.3% for LVO detection (33). The advantage of SONAS BPU lies in the simplicity of simultaneous hemispheric perfusion measurements after bolus injection of EEA, which may be carried out by paramedics with telemedical support.

The aim of the present study was to analyze the SONAS study patient dataset to compare the sensitivity and specificity of various prehospital stroke scores and the SONAS device for LVO detection, and to assess whether combining BPU with prehospital scores enhances diagnostic confidence in identifying acute LVO.

## Methods

This study was a secondary analysis of data from the initial SONAS study, with the addition of calculated stroke scales from the initial NIHSS and ultrasound data derived from the patient chart (33). The SONAS study was approved by the local ethics committee of the university clinic Regensburg (21-2,586-104), registered in the European union clinical trials register (EudraCT Number: 2018-001279-19) and the final patient was enrolled December 19th 2019. No new data was acquired for this study.

In brief, the SONAS study investigated whether a novel non-imaging ultrasound device was able to detect hemispheric perfusion differences in patients with acute ischemic stroke. The SONAS^®^ device is a battery-powered, non-imaging ultrasound system weighing only 2.2 kg, consisting of a transducer-headset that is placed over the patient’s temporal sound windows like headphones, and an operator unit. After injection of a FDA-approved ultrasound contrast agent (UCA, Sonovue^®^), an increased backscatter leading to hemispheric time intensity curves that correlate to brain perfusion, as previously demonstrated by Kilic et al. (34) with perfusion MRI. The time between injection and maximum signal is referred to as the ‘time to peak’ (TTP). Any changes in the hemispheric TTP are displayed as a numerical value, e.g., LVO of the middle cerebral artery would result in delayed or absent hemispheric contrast inflow. The results (LVO/non-LVO) of the SONAS measurement of the 20 study patients performed immediately after admission were compared with CT perfusion imaging (32). Briefly, inclusion criteria were age ≥ 18 years, with a clinical diagnosis of ischemic stroke (NIHSS > 6 and exclusion of intracerebral hemorrhage (ICH)), onset of symptoms <24 h and assessment of brain perfusion using perfusion cCT. Main exclusion criteria included existing contraindications to the SonoVue^®^, severe cardiac or pulmonary disease, acute endocarditis and artificial heart valve, amongst others. However, none of these occurred in the study population. Fourteen of the 20 patients included were female (70%). All patients were of Caucasian origin. The patients ranged in age from 44 to 91 years, with an average age of 72.8 years. For each patient included, NIHSS certified doctors acquired the NIHSS within 30 min after admission. MCAO was present in 17 (85%) of the 20 patients according to the reference standard. The SONAS assessment could not be performed in two subjects due to low data quality. Of the 18 evaluable datasets, 14 subjects were correctly identified with existing MCAO by the SONAS assessment and 2 were correctly identified with no MCAO. However, a single patient was identified as having a perfusion deficit by the SONAS assessment that was not detected as stroke by the reference standard. An additional subject was identified as not having a perfusion deficit by the SONAS assessment but was confirmed as having stroke as detected by the reference standard (33). Since SONAS was a single center trial in our clinic, we had access to all patient source data.

### Prehospital stroke scales

The acquired NIHSS data of the SONAS study participants were used to retrospectively calculate 16 different prehospital scores, of which 10 of them had NIHSS equivalences described in the validation studies: RACE, CPSSS, CPSS and d-CPSS, VAN, PASS, FAST-ED, CG-FAST, G-FAST, and sNIHSS-EMS (see Table 1, see Supplementary material). In addition, we investigated the following stroke scales with items that were not directly transferable from the NIHSS: LAMS, LARIO, 3I-SS, ELVO, VES, and M-DIRECT (see Table 2). Of these scores, G-FAST, CG-Fast, RACE and NIHSS perform best according to a recent prospective study by Duvekot in 2021 (28).

**Table 1:** Translation of prehospital stroke scores from the NIHSS as described in the validation studies (for detailed explanation see Supplementary material).

NIHSS	Stroke scales	LAMS	LARIO	3I-SS	ELVO	VES	M-DIRECT	pH-ICH
1a. Level of consciousness	0 = Alert; keenly responsive			0		Negative		0
	1 = Not alert, but arounsable by minor stimuation			1		Positive		1
	2 = not alert; requires repeated stimulation			2		Positive		2
	3 = Unresponsive or responds only with reflex			2		Positive		3
1b. What is the month/age?	0 = both answers correct				Negative			
	1 = Answers 1 question correctly				Positive			
	2 = Answers 2 questions incorrectly				Positive			
1c. Open,close eyes / grip,release hand	0 = both tasks correctly					Negative	0	0
	1 = 1 task correctly					Negative	1	1
	2 = 2 tasks incorrectly					Positive	2	2
2. Best gaze	0 = normal			0	Negative	Negative		
	1 = Partial gaze palsy			1	Positive	Positive		
	2 = Forced deviation			2	Positive	Positive		
3. Visual	0 = no visual loss				Negative			0
	1 = partial hemianopia				Positive			1
	2 = complete hemianopia				Positive			2
	3 = Bilateral hemianopia				Positive			3
4. Facial palsy	0 = normal symmetric movements	0	0				0	
	1 = minor paralysis	0	1				1	
	2 = partial paralysis	1	1				2	
	3 = complete paralysis of 1 or both sides	1	1				2	
5. Motor arm right or left	0 = no drift	0 /grip strength 0	0 / grip strength 0	0			0	0
	1 = drift	1 / grip strength 1	1 / grip strength 1	1			0	1
	2 = some effort against gravity	2 /grip strength 1	1 / grip strength 1	2			1	2
	3 = no effort against gravity;limb falls	2 /grip strength 2	1 / grip strength 1	2			2	3
	4 = no movement	2 / grip strength 2	1 / grip strength 1	2			2	4
6. Motor leg right or left	0 = no drift						0	0
	1 = drift						0	1
	2 = some effort against gravity						1	2
	3 = no effort against gravity						2	3
	4 = no movement						2	4
7. Limb ataxia	0 = absent						0	
	1 = Present in 1 limb						1	
	2 = Present in 2 limbs						1	
8. Sensory	0 = Normal							0
	1 = mild to moderate sensory loss							1
	2 = severe to total sensory loss							2
9. Best language	0 = no aphasia		0		Negative	Negative		
	1 = Mild to moderate aphasia		1		Negative	Positive		
	2 = severe aphasia		1		Positive	Positive		
	3 = mute, global aphasia		1		Positive	Positive		
10. Dysarthria	0 = normal		0		Negative			
	1 = mild to moderate dysarthria		1		Negative			
	2 = severe dysarthria		1		Positive			
11. Extinction and inattention	0 = no abnormality		0			Negative	0	
	1 = visual, tactile, auditory, spatial, personal inattention		1			Positive	1	
	2 = Profound hemi-inattention or extinction		1			Positive	2	
Systolic bloodpressure + age + RACE	< 190 mmHg / <81a / ≥ 5						Yes = positiv	
Systolic bloodpressure	≥ 180 mmHg							1
LOC	1a or 1b or 1c ≥ 1							1

**Table 2:** List of translations of prehospital stroke scores that cannot be transferred directly from the NIHSS (for detailed explanation see Supplementary material).

NHISS	Stroke scales	RACE	CPSSS	CPSS	d-CPSS	VAN	PASS	FAST-ED	CG-FAST	G-FAST	sNIHSS-EMS
1a. Level of consciousness	0 = Alert; keenly responsive										0
	1 = Not alert, but arounsable by minor stimuation										1
	2 = not alert; requires repeated stimulation										2
	3 = Unresponsive or responds only with reflex										3
1b. What is the month/age?	0 = both answers correct		0				No		0		
	1 = Answers 1 question correctly		1				Yes		1		
	2 = Answers 2 questions incorrectly		1				Yes		1		
1c. Open,close eyes / grip,release hand	0 = both tasks correctly	0	0								
	1 = 1 task correctly	1	1								
	2 = 2 tasks incorrectly	2	1								
2. Best gaze	0 = normal		0				No	0	0	0	
	1 = Partial gaze palsy		2				Yes	1	1	1	
	2 = Forced deviation		2				Yes	2	1	1	
3. Visual	0 = no visual loss					No					
	1 = partial hemianopia					Yes					
	2 = complete hemianopia					Yes					
	3 = Bilateral hemianopia					Yes					
4. Facial palsy	0 = normal symmetric movements	0		0	0			0	0	0	0
	1 = minor paralysis	1		1	1			0	1	1	1
	2 = partial paralysis	2		1	2			1	1	1	2
	3 = complete paralysis of 1 or both sides	2		1	3			1	1	1	3
5. Motor arm	0 = no drift	0	0	0	0	No	No	0	0	0	0
	1 = drift	0	0	1	1	Yes	Yes	1	0	0	1
	2 = some effort against gravity	1	1	1	2	Yes	Yes	1	1	1	2
	3 = no effort against gravity;limb falls	2	1	1	3	yes	Yes	2	1	1	3
	4 = no movement	2	1	1	4	Yes	Yes	2	1	1	4
6. Motor leg	0 = no drift	0									0
	1 = drift	0									1
	2 = some effort against gravity	1									2
	3 = no effort against gravity	2									3
	4 = no movement	2									4
7. Limb ataxia	0 = absent	0									
	1 = Present in 1 limb	1									
	2 = Present in 2 limbs	1									
8. Sensory	0 = Normal										0
	1 = mild to moderate sensory loss										1
	2 = severe to total sensory loss										2
9. Best language	0 = no aphasia			0	0	No		0	0	0	0
	1 = Mild to moderate aphasia			1	1	Yes		1	1	1	1
	2 = severe aphasia			1	2	Yes		2	1	1	2
	3 = mute, global aphasia			1	3	Yes		2	1	1	3
10. Dysarthria	0 = normal			0	0				0	0	0
	1 = mild to moderate dysarthria			1	1				1	1	1
	2 = severe dysarthria			1	2				1	1	2
11. Extinction and inattention	0 = no abnormality	0				No		0			
	1 = visual, tactile, auditory, spatial, personal inattention	1				Yes		1			
	2 = Profound hemi-inattention or extinction	2				Yes		2			

The SONAS trial excluded patients with ICH, the main differential diagnosis in severe acute ischemic stroke, as its primary aim was to evaluate accuracy in detecting perfusion asymmetry. In this secondary analysis, we performed an exploratory analysis using the prehospital intracerebral hemorrage (pH-ICH) score that was validated in the mobile stroke unit project in Berlin in ICH analysis only in order to compare this to the BPU results (35).

In all scores we used the published cutoff values that yielded the highest sensitivity and specificity (see Supplementary material).

### Statistical analysis

The various prehospital stroke scores, SONAS results and the combination of prehospital stroke scores with SONAS BPU results were applied to the 20 study patients and their sensitivity, specificity, positive and negative predictive value (PPV and NPV) were calculated with regard to their recognition of LVO.

## Results

### Comparison of the performance of different prehospital scales

In the SONAS study population, patients were classified into two groups according to the initial CT results (which included CT-Angiography and CT-Perfusion): “large vessel occlusion present” (n = 17) and “large vessel occlusion absent” (n = 3). The results of the cerebral imaging were used to define the “ground truth,” on which all further analyses are based on.

We focused our primary analysis on the scores found to perform best: G-FAST, CG-Fast, RACE, and NIHSS, while the results of the other analyzed scores are shown in the Supplementary material (Figure 1; see also Supplementary material). Overall, these four scores showed good results: With a cut-off between 2 and 3, G-FAST as well as CG-FAST would have missed an LVO in a single patient (#20), while correctly classifying all other patients. This corresponds to a sensitivity of 94% (16/17) and a specificity of 100% (3/3). The PPV would be 100% (16/16) and the NPV would be 75% (3/4). Though the performance of the two scores was identical regarding sensitivity, specificity, PPV and NPV, CG-FAST should be preferred based on our results as it seems to be able to classify patients better (the “C”-aspect of the score was only added to patients with LVO and thus the group means have a larger distance) (22).

**Figure 1:**
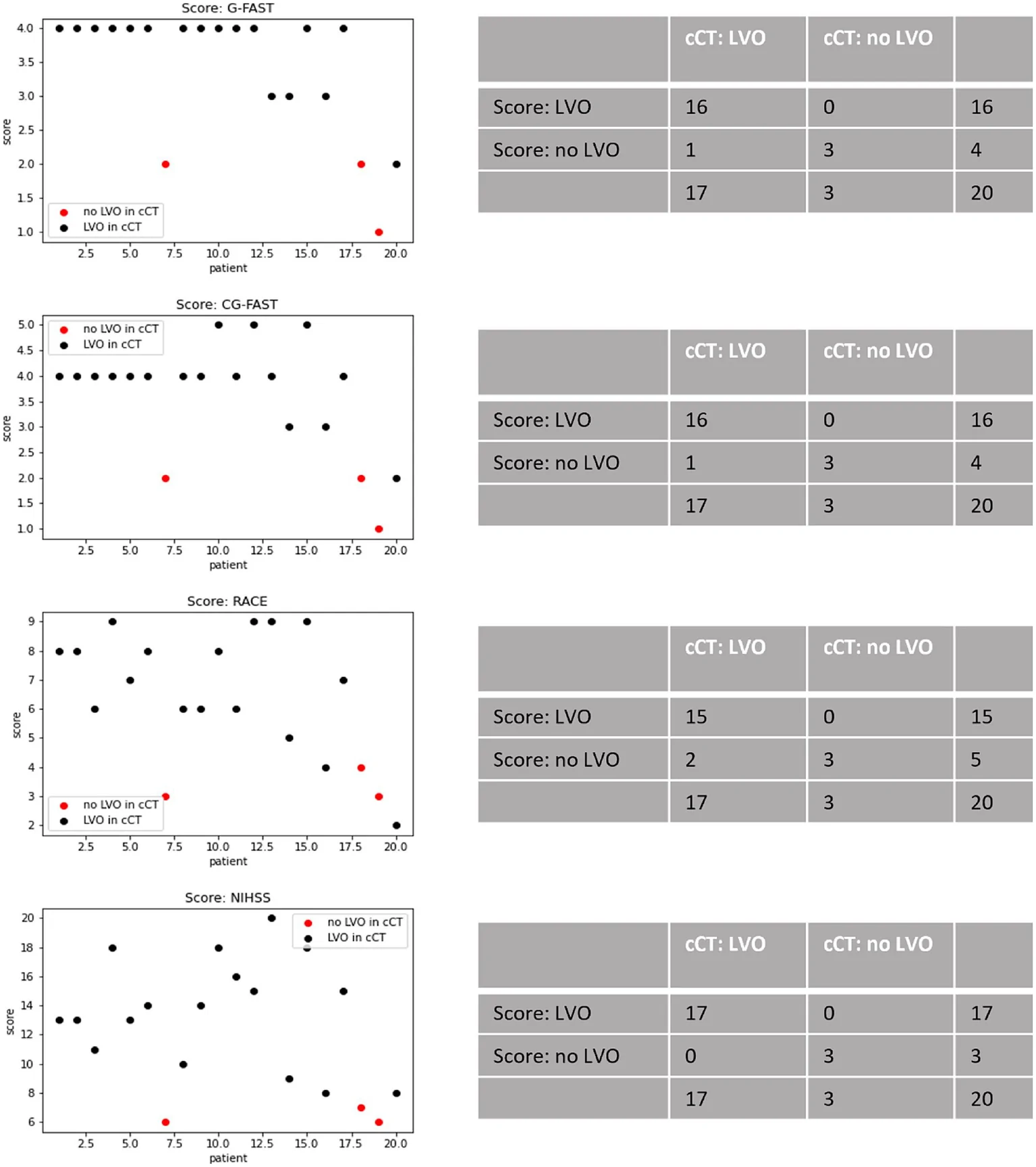
Performance of different scores within our cohort. Black dots represent patients with large vessel occlusion, red dots those without LVO. Cut-off values for the classification of LVO vs. non-LVO based on the scores were set between 2 and 3 for G-FAST and CG-FAST, between 4 and 5 for RACE and between 7 and 8 for the NIHSS. The tables next to the images show the respective contingency tables.

With regard to the RACE score, the cut-off value was set between 4 and 5. This classification would have missed two patients with LVO (#16 and #20) and would have classified all other patients correctly. This corresponds to a sensitivity of 88% (15/17), a specificity of 100% (3/3), PPV of 100% (15/15), and an NPV of 60% (3/5).

While G-FAST, CG-FAST and RACE are common prehospital scores, the NIHSS is the most common hospital score used to assess stroke severity, including in patients with LVO. If the cut-off value would have been set between 7 and 8, it would have been able to classify all of our patients within the cohort correctly. This would correspond to a sensitivity, specificity, PPV and NPV of 100% each.

### Performance of SONAS

The SONAS-BPU device was unable to demonstrate time-intensity curves in 2 out of 20 patients (#6 and #17) in the SONAS study (33). The device indicated low data quality, due to either high attenuation of the ultrasound from inadequate temporal bone windows, decreased signal-to-noise ratio or non-optimal headset placement. In the remaining 18 patients, the data showed a very good concordance with the CT results as illustrated in the contingency table (Table 3). This corresponds to a sensitivity of 93% (14/15), a specificity of 66% (2/3), a PPV of 93% (14/15), and an NPV of 66% (2/3).

**Table 3:** Contingency table comparing the performance of our mobile ultrasound device with the “ground truth” defined by cerebral computed tomography.

SONAS test	Confirmed stroke diagnosis (cCT)		Total
	Positive	Negative	
Positive	14	1	15
Negative	1	2	3
Total	15	3	18

### Combination of BPU-SONAS with prehospital scales

To investigate whether or not the combination of the two best permorming prehospital scales (C(G)-FAST, RACE) and BPU improved LVO detection, we analyzed the following scenarios: If both examinations showed negative results, the patient was classified as “no large vessel occlusion”. If both showed positive results, the patient was classified as “large vessel occlusion”. For contradicting analyses, there were two possibilities: (A) classification as “large vessel occlusion” or (B) classification as “no large vessel occlusion” (Figure 2). For these contradicting analyses, Scenario (A), or empiric classification as “LVO,” should be chosen if an increase in sensitivity is wished for at the cost of specificity, while Scenario (B), or determination of “no LVO,” would lead to an increase in specificity and a decrease in sensitivity. Scenario (A) would favor a good outcome in patients with real LVO at the cost of patients with smaller vessel occlusion or stroke mimics (as the “unclear” patients with contradicting predictions are transferred to the next hospital capable of mechanical thrombectomy), Scenario (B) would favor patients with small vessel occlusions and stroke mimics that need urgent medical treatment (as the “unclear” patients are transferred to the nearest hospital). To perform these analyses, the two patients that did not have useable data from SONAS were excluded, reducing the total number of patients to 18.

**Figure 2:**
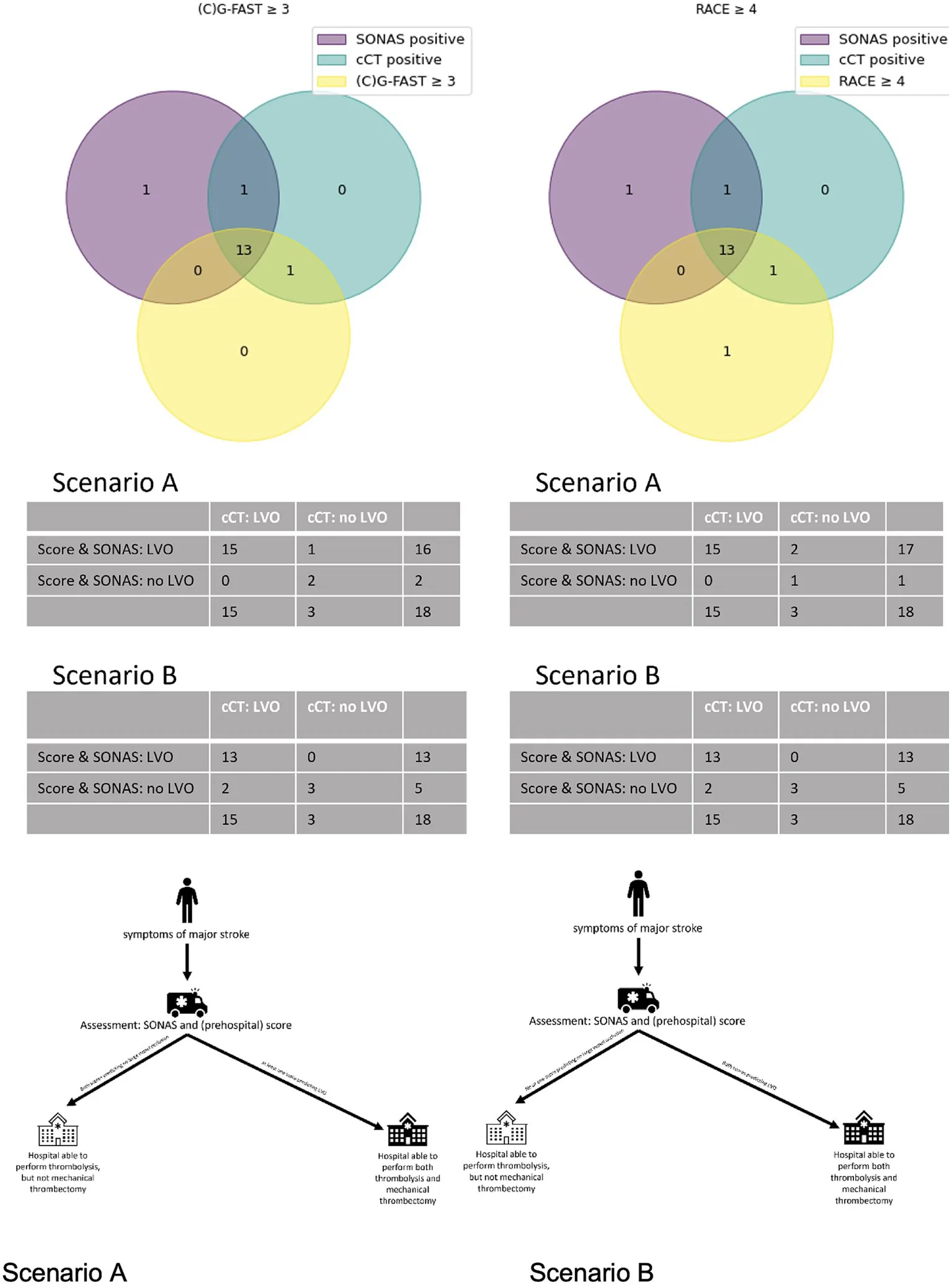
Results of the combination of the mobile ultrasound prediction with the prehospital scores G-FAST/CG-FAST and RACE. The upper two figures show Venn diagrams of patients in the SONAS study, excluding those with negative predictions and no LVO on CT assessment (*n* = 2 in the FAST-Scores and *n* = 1 in RACE). Below, the corresponding contingency tables are shown for the two different scenarios as described in the text. At the bottom, the two scenarios are shown as graphical schemes.

### Scenario A

#### (C) G-Fast

The sensitivity of the combined method was 100% (15/15), the specificity 66% (2/3), the PPV 94% (15/16), and the NPV 100% (2/2). Compared to the results of the score alone, the sensitivity would have increased (score alone 94%) at the cost of specificity (score alone 100%). The PPV would have also decreased (score alone 100%) with an increase in negative predictive value (score: 75%). Overall, a shift of the prediction values would have been achieved without clear improvement or deterioration.

#### Race

The same applies to the RACE score in scenario A. The sensitivity would increase from 88 to 100% at the cost of specificity with a decrease from 100 to 33%. The PPV would decrease from 100 to 88% and the NPV would have increased from 60 to 100%.

### Scenario B

#### (C) G-Fast

The sensitivity of the combined method would be 86% (13/15), specificity 100% (3/3), PPV 100%, and the NPV 60% (3/5). Compared to the results of the prehospital score alone, the values remained almost identical.

#### Race

The sensitivity of the combined method would be 86% (13/15), specificity 100% (3/3), PPV 100%, and the NPV 60% (3/5). Again, compared to the results of the prehospital score alone were almost identical.

### Scenario C

Scenario C examines the situation in patients that are LVO-positive by SONAS, but not by the prehospital score, or vice versa. In this scenario, patients are grouped into three clusters: “clear LVO,” “clearly no LVO” and “unclear.” This scenario is shown in Figure 3 and makes maximal use of both the advantages of the SONAS prediction (high sensitivity) and the score prediction (high specificity).

**Figure 3:**
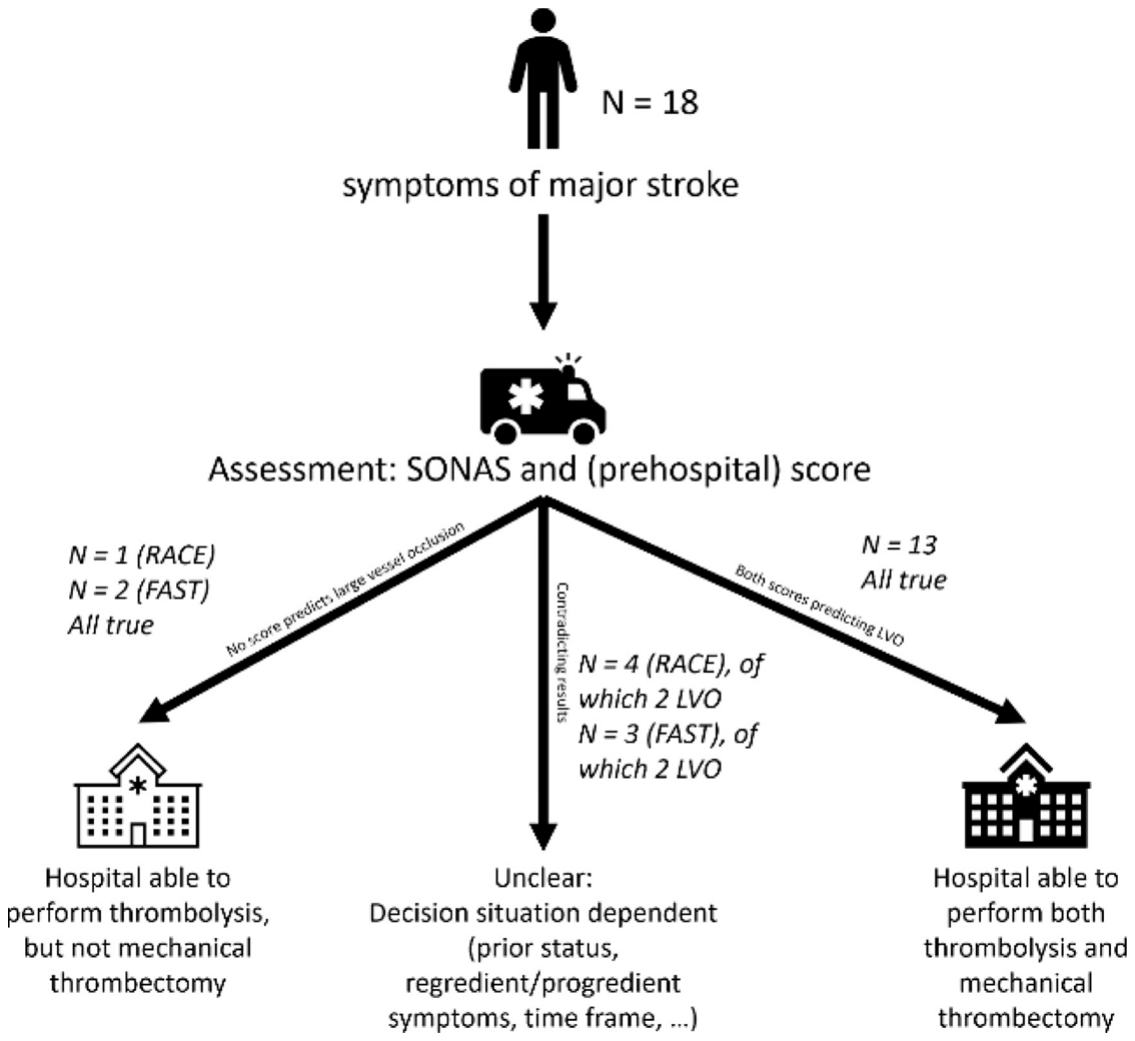
Scenario C with three instead of two possible classifications. In this scenario, patients are grouped into “clear LVO” if SONAS-BPU and the prehospital score both predict a large vessel occlusion. These patients are transferred to the nearest hospital capable of mechanical thrombectomy. If both predictions are negative, the patients are classified as “clearly no LVO” and transferred to the nearest hospital capable of thrombolysis and thus do not lose further time by being transported to a hospital in a longer distance. Patients that have contradicting predictions are classified into a third group, “unclear cases,” without a strictly defined procedure. This classification might also be named “possible LVO.” Depending on the available resources, difference in transportation time, pre-morbid mRS, evolution of symptoms (progressing vs. improving), as well as time frame (early presentation vs. presentation after about 4 h), a more informed decision can be made for these patients.

This combination of the scores would have classified either 1 patient (by RACE score) or 2 patients (by CG-FAST and G-FAST scores) as “no LVO,” respectively. Both classifications would have been correct. The same applies to 13 patients in which both BPU-SONAS as well as the three prehospital stroke scores classified the patients into “clear LVO” – again, all classifications would have been correct. Thus, this classification system increases the predictive values.

The classification of “unclear” cases, in which an LVO might be present but contradicting predictions have emerged, would have been recognized reliably. This would have occurred in 4 patients using RACE (with 2 having a real LVO) and 3 patients using (C)G-FAST, of which 2 had an LVO. Most importantly, this group included ALL patients with false predictions by either the prehospital score or BPU-SONAS, demonstrating this classification system as highly effective in recognizing unclear or falsely classified cases.

In an exploratory analysis we applied the only validated prehospital intracerebral hemorrhage score (pH-ICH) to our patient population with cerebral ischemia only (35). A pH-ICH score of <3 indicates that ICH is more likely than cerebral ischemic stroke, and this constellation was found in 3 of our 20 patients (false positive results). In all of these 3 patients, a perfusion deficit could be detected in both SONAS-BPU and perfusion CT.

## Discussion

In this study, we investigated the performance of 16 different prehospital stroke scales on their ability to detect LVO in a pre-selected population of acute ischemic stroke patients with and without LVO enrolled in the SONAS-Trial (33). The two most promising stroke scores, (C)G-FAST and RACE, were also analyzed in conjunction with the results of brain perfusion ultrasound by the SONAS device. In summary, the simple combination of SONAS with prehospital stroke scores does not add to the predictive values themselves but enables adjustment for preference of sensitivity over specificity, PPV over NPV, and vice versa. Accurate diagnosis of either LVO or ICH is critical for site-specific transfer and appropriate stroke treatment, balancing the benefit of early reperfusion in LVO patients with the risk of potentially delayed IVT in non-LVO patients or delayed initiation of antihypertensives and reversal agents in ICH patients.

Since the expansion of recanalization therapies for the treatment of acute ischemic stroke, prehospital identification and separation of stroke mimics has been the primary goal of clinical prehospital scores (36). The FAST-MAG trial was the first prehospital interventional trial making use of a prehospital stroke identification score – FAST (37). In the study population, acute ischemic stroke comprised 73.3% of patients, while ICH was present in 22.8%, and a stroke-mimicking condition in 3.9%. Since then, a variety of prehospital stroke scales have been designed and tested with the primary goal of clinically identifying LVOs in the field. Yet, many were tested in selected populations, i.e., in known ischemic stroke patients, excluding those with ICH, by medical doctors in the emergency ward or stroke unit, or in registries with IVT and MT patients only (22, 27). The RACE score is one of the few scores to have been tested prior the clinical RACECAT trial (15, 18). However, according to the first study, 20% of patients in the RACECAT were re-directed to CSCs for MT actually suffered from ICH. Interestingly, in the RACECAT study, patient transport to the nearest stroke unit with suspected LVO led to better chances of functional independence at 90 days for patients with ICH (38). This positive effect may be attributed to earlier hospital arrival of patients with earlier CT diagnosis of ICH, initiation of early blood pressure management, prevention of aspiration, control of seizures, and with hemostatic normalization in patients with anticoagulation. In summary, bypassing PSCs for suspected LVO has the potential of backfiring in patients with ICH, and calls for earlier prehospital stroke diagnostics. Even the addition of machine learning algorithms to prehospital stroke scales was found to be imperfect due to a lack of external validation in randomized studies (39). Therefore, if a single clinical score that can detect LVO preclinically with both high sensitivity and high specificity is not available, a scoring system to identify ICH may help (40, 41). A recent meta-analysis by Almubayyidh et al. (40, 41) evaluated the performance of prehospital models in distinguishing ICH from other causes of suspected stroke, identifying 9 prediction studies, five of which were designed to differentiate between stroke subtypes and three to distinguish between ICH and acute ischemic stroke-- including the study by Geisler et al. defining pH-ICH, which was specifically designed to identify ICH and therefore used in our study to test for false-positive results (35, 41). In the meta-analysis, all studies were assessed as having a high risk of bias, particularly in the analysis domain. The performance of the models varied, with the area under the receiver operating characteristic curve ranging from 0.73 to 0.91.

The current study, which used the highly selected population of the SONAS trial lacking patients with ICH (as the SONAS trial was primarily designed to test patients with relevant hemispheric perfusion differences), identified the (C)G-FAST and RACE scores as having high sensitivity and specificity to detect LVO patients. In addition to being a feasibility study with only 20 patients, the main limitations of this study include the lack of randomization and blinding, application by trained stroke neurologists not paramedics or emergency physicians, as well as the exclusion of patients with stroke mimics, including those with ICH or subarachnoid hemorrhage. Application of the ph-ICH score the SONAS study cohort incorrectly classified 15% of patients (3 of 20 patients) as having ICH over ischemic stroke similar to the misclassification in the RACECAT study of ICH as LVO by the application of the RACE score (18, 38). Thus, our results are very much in line with previous comparative studies on prehospital stroke scales, supporting the overall validity of the results (15, 42). However, in all 3 both perfusion CT and BPU correctly identified significant perfusion deficits. Indeed, little data exist on hemispheric perfusion abnormalities in ICH, and none have been published with the SONAS™-device yet. Few studies examining perfusion changes in patients with spontaneous ICH focused on local perfusion changes round the ICH (43). However, the observed changes are minor compared to LVO: In a recent study in ICH patients without midline shift the mean perfusion deficit on single photon emission tomography (SPECT) was 15% only perilesionally, attributed to compression of the neighboring brain tissue by perihematoma oedema (44).

In order to improve prehospital stroke diagnosis and diagnostic confidence, especially in severe stroke such as ICH and LVO, additional diagnostic testing seems mandatory to further identify, characterize and stratify stroke-call patients in the prehospital phase that are unselected and investigation should be performed by trained paramedics or emergency doctors perhaps with telemedical support by experienced stroke neurologists. This ancillary testing may be classified as blood-serum based or imaging-neurophysiological based, and with or without the aid of telemedicine. Blood-serum biomarkers may include NSE, MMP-8, S-100B, D-Dimer, GFAP, amongst others, and technical devices for cerebral imaging and functional assessment may be microwave-based, mobile MRI-units, mobile electroencephalography and evoked potentials, and neurosonography, amongst others (45–50). Measurement of glial fibrillary acidic protein may be the serum biomarker best characterized in the prehospital field and with the greatest potential to identify ICH, though its use limited to the first hours after symptom onset (51–53). Mobile stroke units are able to combine a variety of these aforementioned techniques, but their limited mobility range may restrict use to more densely populated areas (54–56). Our own group had prehospital experience with conventional portable transcranial color-coded ultrasound, and experienced neurologists achieved high sensitivity and specificity of >90% in identification of MCA occlusions (31, 32). However, deployment of stroke neurologists for pre-hospital stroke diagnostics is inefficient and requires great individual enthusiasm and funding.

### Outlook

If diagnostic confidence in LVO identification is sufficiently high, it could pave the way for prehospital thrombolysis. Recently, tenecteplase has been approved as an alternative and potentially superior thrombolytic agent than alteplase in acute ischemic stroke, and especially in patients with LVO (57, 58). Given its ease of administration, tenecteplase would be the ideal agent to perform prehospital thrombolysis in patients for whom there is high diagnostic confidence of acute ischemic stroke from SONAS-BPU and prehospital stroke scales, possibly with exclusion of ICH using serum GFAP and confirmatory support by stroke specialists via telemedicine (41). Another consequence of this combined use of SONAS-BPU with RACE or (C)G-FAST may be the proper adjustment of blood-pressure and careful revereal of ongoing anticoagulation in the prehospital setting. The importance of this was suggested by the recent INTERACT-4 trial, in which blood-pressure lowering may be beneficial in ICH but harmful in ischemic stroke (59). Also, accurate prehospital LVO identification may further aid concept such as “direct to angio-suite” approaches (4, 60). Technical refinement of the SONAS™ device for detection of posterior circulation ischemic stroke (especially acute posterior cerebral artery and basilar artery occlusion), presently underway, are urgently needed, as this population was absent in the current study and pose additional challenges in diagnosis and prognosis.

As stroke is a time critical disease, any time delay using POC diagnostics including stroke scales, use of MSU and SONAS™ should be as brief as possible. Thus, comparative studies between various methods of POC equipment for stroke diagnostics should include timelines, sensitivity/specificity, overall costs, simplicity and robustness, and should be performed prehospital arrival in an unselected stroke cohort, including patients ICH, LVO, lacunar stroke, subdural hematoma and stroke mimics, amongst other (61).

## Conclusion

In the present retrospective study, we compared the sensitivity, specificity, and predictive values of various prehospital stroke scales with the novel brain perfusion ultrasound device SONAS in detecting LVO and ICH. While the stroke scales alone demonstrated effectiveness in identifying LVO, our results suggest that combining SONAS-BPU with the highest-performing scales, such as CG-FAST, may contribute to a more efficient framework to guide site-specific transfer strategies for acute stroke patients. However, these findings require validation in larger, more heterogeneous cohorts, including real-world prehospital application by paramedics of both CG-FAST and SONAS-BPU, ideally supported by telemedical support from experienced stroke neurologists.

## Data Availability

The raw data supporting the conclusions of this article will be made available by the authors, without undue reservation.
