# Supplementary material for: Added value of brain perfusion ultrasound to prehospital stroke scales-a secondary analysis of the SONAS trial
Source: Front Neurol. 2026 Oct 23;17:1924664. doi: 10.3389/fneur.2026.1924664 (PMC13739520; doi:10.3389/fneur.2026.1924664)
Supplement: Supplementary file 1 [file Supplementary_file_1.docx]

**Supplement**

The RACE scale was formed from a combination of 5 items from the NIHSS that had the best correlation to LVO detection: 4 (facial palsy), 5 (arm motor function), 6 (leg motor function), 2 (gaze) and 9 (aphasia) or 11 (neglect) with respective ratings of severity between 0 and 2 points according to the validation study table ^1^.

The CPSSS contains the NIHSS items 2 (gaze) with a score of 2 points, 1b and 1c (LOC questions and commands) and 5 (arm motor funktion) with a score of 1 point each as soon as a certain NHISS score is exceeded ^2^.

The CPSS is scored from 0 to 3, with one point given for each of the following physical exam findings: facial drop (NIHSS item 4), arm drift (NIHSS item 5), and slurred speach (NIHSS item 9 or 10) ^3^.

The detailed d-CPSS contains the same items as the CPSS, but has the same scoring system as the NIHSS ^4^.

VAN includes the NIHSS items 5 (arm weakness), 3 (visual disturbance), 9 (aphasia) and 11 or 2 (neglect and gaze). These are either present or absent if the NIHSS in the respective category is >0 ^5^.

The PASS targets 3 items of the NIHSS. These are 1b (orientation), 2 (gaze) and 5 (arm weakness). These 3 items are also rated as either positive or negative if the NIHSS score is >0 ^6^.

FAST-ED contains the NIHSS items 4 (facial palsy), 5 (arm weakness), 9 (aphasia), 2 (gaze) and 11 (neglect). The points were assigned according to the table of the validation study ^7^.

CG-FAST includes the NIHSS items 1b (orientation), 2 (gaze), 4 (facial palsy), 5 (arm weakness) and 9 (aphasia) with 1 point for each item if the NIHSS >0 ^8^.

With the G-FAST, the item gaze is added to the FAST score. It includes the NIHSS items 2 (gaze), 4 (facial palsy), 5 (arm weakness) and 9 (aphasia) with 1 point for each item if the NIHSS >0 ^9^.

The NIHSS-EMS is a shorter form of the NIHSS, which is limited to a selection of 7 items, which remain the same with their scoring. The selection consists of: 1b (orientation), 4 (facial palsy), 5 (arm weakness), 6 (leg weakness), 8 (sensory), 9 (aphasia), 10 (dysarthria) ^10^.

For LAMS, the items Facial drop and Arm drift were taken from the NIHSS. The grip strength was determined as follows, taking into account the method section of the validation study: Normal 0 -> NIHSS 0 / weak grip 1 -> NIHSS 1-2 / no grip -> NIHSS 3-4 ^11^.

A similar procedure was followed for LARIO. Clear adoption of the items facial palsy, arm weakness, language and neglect from the NIHSS. Grip strength reduced for NHISS item 5 = 1-4 ^12^.

The 3I-SS was derived from the NIHSS using the method section of the validation study as follows: disturbance of consciousness: no -> NIHSS 0 / mild -> NIHSS 1 / severe -> NIHSS 2-3; gaze and head deviation: absent -> NIHSS 0 / incomplete -> NIHSS 1 / forced -> NIHSS 2; hemiparesis: absent -> NIHSS 0 / moderate -> NIHSS 1 / severe -> NIHSS 2-4 ^13^.

In the ELVO the eye position was taken from NIHSS Item 2 (gaze) (NIHSS>0 = positive). The questions ‘What is this?’ show glasses or watch or How many fingers? 4, are not included in the NIHSS. The NIHSS items 1b and 3 were translated as the most comparable with NIHSS 1b>0 = positive and NIHSS 3 >0 = positive. In addition, items 10 (dysarthria) and 9 (aphasia) were considered if they contained NIHSS values ≥ 2 ^14^.

In the ‘forced deviation of both eyes’ section, the VES also only offers approximate comparability in the NIHSS (decision: positive from NIHSS 2 oculomotor function = 1 point), better for aphasia (decision: positive if NIHSS 9 aphasia = 1-3 points / NIHSS 1a = 0 / NIHSS 1c = 2), whereby the VES also requires object naming here, which must be disregarded. In addition, the consideration of dysarthria should also be discussed here (although this makes no difference in the evaluation). The neglect and sopor items can be easily determined from the NIHSS. (Neglect positive if NIHSS 11 = 1-2 points, sopor (decision: positive if NIHSS 1a = 1-3 points) ^15^.

The M-DIRECT consists of the items of the RACE ≥ 5 as well as a systolic bloodpressure <190mmHg and age < 81a. The RACE score was derived from the NIHSS as described above; age and systolic blood pressure on admission were available from the SONAS study ^16^.

The pH-ICH is the only one of these scores that detects cerebral hemorrhage rather than cerebral vascular occlusion. In addition to neurological deficits, which all correspond to the NIHSS items, systolic blood pressure ≥ 180mmHg is also included. These data were also available as part of the SONAS study ^17^.

**List of Abbreviations in order of appearance**

| EVT | endovascular thrombectomy |
| --- | --- |
| LVO | large vessel occlusion |
| CSC | comprehensive stroke centers |
| IV | intravenous |
| MT | mechanical thrombectomy |
| PSC | primary stroke center |
| QALYs | quality adjusted life years |
| IVT | Intravenous thrombolysis |
| CT | computertomography |
| EEG | electroencephalography |
| TCD | transcranial Doppler |
| TCCS | Transcranial color coded sonography |
| EEA | echo enhancing agents |
| MCAO | middle cerebral artery occlusion |
| i.e. | it est = that means |
| BPU | brain perfusion ultrasound |
| NIHSS | National Institutes of Health Stroke Scale |
| FDA | Food and Drug Administration |
| UCA | ultrasound contrast agent |
| MRI | magnetic resonance imaging |
| TTP | time to peak |
| e.g. | exempli gratia = for example |
| ICH | intracerebral hemorrhage |
| h | hour |
| cCT | cerebral computer tomography |
| RACE | Rapid Arterial oCclusion Evaluation |
| CPSSS | Cincinnati Prehospital Stroke Severity Scale |
| CPSS | Cincinnati Prehospital Stroke Scale |
| d-CPSS | detailed Cincinnati Prehospital Stroke Scale |
| VAN | Vision Aphasia Neglect |
| PASS | Prehospital Acute Stroke Severity |
| FAST-ED | Field Assessment Stroke Triage for Emergency Destination |
| CG-FAST | Conveniantly Grasped Field Assessent Stroke Triage |
| G-FAST | Gaze Field Assessment Stroke Triage |
| sNHISS-EMS | shorted NIHSS for Emergency Medical Services |
| LAMS | Los Angeles Motor Scale |
| LARIO | Large ARtery Intracranial Occlusion |
| 3I-SS | 3 Item Stroke Scale |
| ELVO | Emergent Large Vessel Occlusion |
| VES | Ventura Emergent large vessel occlusion Score |
| M-DIRECT | Madrid DIrect Referral to Endovascular CenTer |
| pH-ICH | prehospital intracerebral hemorrhage |
| PPV | positive predictive value |
| NPV | negative predictive value |

**References**

1.	Perez de la Ossa N, Carrera D, Gorchs M, et al. Design and validation of a prehospital stroke scale to predict large arterial occlusion: the rapid arterial occlusion evaluation scale. Stroke 2014;45:87-91.

2.	Katz BS, McMullan JT, Sucharew H, Adeoye O, Broderick JP. Design and validation of a prehospital scale to predict stroke severity: Cincinnati Prehospital Stroke Severity Scale. Stroke 2015;46:1508-12.

3.	Hurwitz AS, Brice JH, Overby BA, Evenson KR. Directed use of the Cincinnati Prehospital Stroke Scale by laypersons. Prehosp Emerg Care 2005;9:292-6.

4.	Tarkanyi G, Csecsei P, Szegedi I, et al. Detailed severity assessment of Cincinnati Prehospital Stroke Scale to detect large vessel occlusion in acute ischemic stroke. BMC Emerg Med 2020;20:64.

5.	Teleb MS, Ver Hage A, Carter J, Jayaraman MV, McTaggart RA. Stroke vision, aphasia, neglect (VAN) assessment-a novel emergent large vessel occlusion screening tool: pilot study and comparison with current clinical severity indices. J Neurointerv Surg 2017;9:122-6.

6.	Hastrup S, Damgaard D, Johnsen SP, Andersen G. Prehospital Acute Stroke Severity Scale to Predict Large Artery Occlusion: Design and Comparison With Other Scales. Stroke 2016;47:1772-6.

7.	Lima FO, Silva GS, Furie KL, et al. Field Assessment Stroke Triage for Emergency Destination: A Simple and Accurate Prehospital Scale to Detect Large Vessel Occlusion Strokes. Stroke 2016;47:1997-2002.

8.	Gong X, Chen Z, Shi F, et al. Conveniently-Grasped Field Assessment Stroke Triage (CG-FAST): A Modified Scale to Detect Large Vessel Occlusion Stroke. Front Neurol 2019;10:390.

9.	Scheitz JF, Abdul-Rahim AH, MacIsaac RL, et al. Clinical Selection Strategies to Identify Ischemic Stroke Patients With Large Anterior Vessel Occlusion: Results From SITS-ISTR (Safe Implementation of Thrombolysis in Stroke International Stroke Thrombolysis Registry). Stroke 2017;48:290-7.

10.	Purrucker JC, Hartig F, Richter H, et al. Design and validation of a clinical scale for prehospital stroke recognition, severity grading and prediction of large vessel occlusion: the shortened NIH Stroke Scale for emergency medical services. BMJ Open 2017;7:e016893.

11.	Stead TG, Banerjee P, Ganti L. Real-World Field Performance of the Los Angeles Motor Scale as a Large Vessel Occlusion Screen: A Prospective Muticentre Study. Cerebrovasc Dis 2021;50:543-50.

12.	Vidale S, Arnaboldi M, Frangi L, Longoni M, Monza G, Agostoni E. The Large ARtery Intracranial Occlusion Stroke Scale: A New Tool With High Accuracy in Predicting Large Vessel Occlusion. Front Neurol 2019;10:130.

13.	Singer OC, Dvorak F, du Mesnil de Rochemont R, Lanfermann H, Sitzer M, Neumann-Haefelin T. A simple 3-item stroke scale: comparison with the National Institutes of Health Stroke Scale and prediction of middle cerebral artery occlusion. Stroke 2005;36:773-6.

14.	Suzuki K, Nakajima N, Kunimoto K, et al. Emergent Large Vessel Occlusion Screen Is an Ideal Prehospital Scale to Avoid Missing Endovascular Therapy in Acute Stroke. Stroke 2018;49:2096-101.

15.	Taqi MA, Sodhi A, Suriya SS, et al. Design, Application and Infield Validation of a Pre-Hospital Emergent Large Vessel Occlusion Screening Tool: Ventura Emergent Large Vessel Occlusion Score. J Stroke Cerebrovasc Dis 2019;28:728-34.

16.	Rodriguez-Pardo J, Fuentes B, Alonso de Lecinana M, et al. The Direct Referral to Endovascular Center criteria: a proposal for pre-hospital evaluation of acute stroke in the Madrid Stroke Network. Eur J Neurol 2017;24:509-15.

17.	Geisler F, Wesirow M, Ebinger M, et al. Probability assessment of intracerebral hemorrhage in prehospital emergency patients. Neurol Res Pract 2021;3:1.
